# Exploring links between social identity, emotion regulation, and loneliness in those with and without a history of mental illness

**DOI:** 10.1111/bjc.12358

**Published:** 2022-02-09

**Authors:** Shaun Hayes, Molly Carlyle, S. Alexander Haslam, Catherine Haslam, Genevieve Dingle

**Affiliations:** ^1^ School of Psychology The University of Queensland St Lucia Queensland Australia; ^2^ Lives Lived Well Research Group School of Psychology The University of Queensland St Lucia Queensland Australia; ^3^ Centre for Health Outcomes Innovation and Clinical Education (CHOICE) School of Psychology The University of Queensland St Lucia Queensland Australia

**Keywords:** emotion regulation, loneliness, mental health, social identity, social support

## Abstract

**Objective:**

Emotion regulation and social identity theorizing provide two influential perspectives on loneliness. From an emotion regulation perspective, loneliness is understood as a negative emotional state that can be managed using emotion regulation strategies. A social identity perspective views loneliness as resulting from a loss or lack of important social groups and related identities. This study aimed to explore the relationships between key constructs drawn from both perspectives, with a view to understanding loneliness in adults with and without a history of mental illness.

**Design and Methods:**

Participants (*N* = 875) with a mental illness history (MH Hx, *n* = 217; *M*_age_ = 45 years, 59% female) and without a mental illness history (No MH Hx, *n* = 658; *M*_age_ = 47 years, 48% female) completed a survey comprising measures of group membership and connectedness, emotion regulation strategies, and loneliness.

**Results:**

The MH Hx group reported higher internal affect worsening strategy use and loneliness than those No MH Hx. Hierarchical regressions indicated that the unique contributions of emotion regulation strategies and social identity factors to loneliness were equivalent between the groups. Together, social identity and emotion regulation explained 37% of the variance in loneliness in the No MH Hx subsample and 35% in the MH Hx subsample.

**Conclusion:**

These findings suggest that both emotion regulation and social identity had significant unique contributions to the reported loneliness of people when controlling for demographics and each other in those with and without a history of mental illness. Integration of the two frameworks may provide novel avenues for the prevention and management of loneliness.

**Practitioner points:**

- Individuals with a history of mental illness report more use of internal emotion worsening regulation strategies and greater loneliness than those with no such history, but there were no differences in social identity factors.
- Internal emotion worsening strategies and social support received from others explained the variance in reported loneliness for both those with and without a history of mental illness.
- Internal emotion improving strategies were significant for those with a history of mental illness, while social support given was significant for those without a history of mental illness.
- Screening clients for emotion regulation difficulties, social disconnectedness, and loneliness may provide clinicians with an indication of risk for developing psychological distress/disorders.

## Background

Loneliness has been defined as the subjective, painful emotional state that occurs when there is a perceived discrepancy between a person’s desired and achieved patterns of social interaction (Hawkley & Cacioppo, 2010). It is associated with a higher risk of health conditions such as stroke, depression, dementia, and substance use disorders (Holt‐Lunstad, Smith, Baker, Harris, & Stephenson, 2015; Ingram et al., 2020; Valtorta, Kanaan, Gilbody, Ronzi, & Hanratty, 2016; Wang et al., 2017), and there is increasing recognition that management of these consequences requires a better understanding of loneliness and its antecedents (Lim, Eres, & Vasan, 2020).

Over several decades, attempts to better understand loneliness have led to the development of numerous models to understand this experience. One example is the social needs model that identified six social needs (i.e., attachment, social integration, nurturance, reassurance of worth, sense of reliable alliance, and guidance in stressful situations) that, if unmet, contribute to feelings of loneliness (Weiss, 1974). Another, the cognitive discrepancy model, proposes that loneliness is predominately driven by the perceived difference between desired and achieved patterns of social interaction (Peplau & Perlman, 1982; Perlman & Peplau, 1998). More recently, the identification of loneliness sub‐types has extended discussion around the nature of loneliness as discussed within these models and identified the importance of multiple elements which can be broadly characterized as either ‘social’ or ‘emotional’ (Dykstra & Fokkema, 2007). Social loneliness is the perceived deficiency in the quantity and/or quality of people’s social network structure. In contrast, emotional loneliness is the subjective experience of sadness and distress associated with loneliness despite any existing networks. However, despite being conceptually distinct these social and emotional components have been found to be moderately correlated, suggesting a core of overlapping experiences (Ditommaso & Spinner, 1997; Green, Richardson, Lago, & Schatten‐Jones, 2001; Russell, Cutrona, Rose, & Yurko, 1984; Salo, Junttila, & Vauras, 2020). Thus, to better understand the unique contributions of social and emotional factors to loneliness, the present study drew on prominent theories – emotion regulation and social identity frameworks – to (1) determine distinct measures of emotional and social factors and (2) evaluate their contribution to perceptions of loneliness in a nationally representative sample of people in the United Kingdom, including some with a history of mental illness (and some without a history of mental illness.

### Emotion regulation and loneliness

The presence of an emotional component of loneliness raises questions about how these emotions are regulated. Emotion regulation refers to the use of internal and interpersonal strategies to modulate how we or others feel (Hofmann, Carpenter, & Curtiss, 2016). Correspondingly, emotion dysregulation refers to maladaptive processes that make the emotional experience *worse*: too intense, long‐lasting, or inappropriate for the context (Hofmann, Sawyer, Fang, & Asnaani, 2012; Laddis, 2015). Both internal and interpersonal emotion regulation strategies have been examined in a range of clinical and non‐clinical samples including adolescents and adults with anxiety, mood, and substance use disorders (Berking & Wupperman, 2012; Dingle, Neves, Alhadad, & Hides, 2018; Verzeletti, Zammuner, Galli, & Agnoli, 2016). Though, in the context of loneliness, research has focused primarily on individuals’ engagement in internal emotion dysregulation such as rumination, avoidance, and emotional suppression. For instance, several researchers have shown that maladaptive internal emotion regulation strategies contribute to a sense of loneliness – particularly in populations experiencing mental health problems (Eres, Lim, Lanham, Jillard, & Bates, 2020; Kearns & Creaven, 2017). Further, a recent study using latent profile analysis identified several loneliness profiles (Preece et al., 2021). Among these, the high loneliness profile was differentiated from others by higher rumination, catastrophizing, and other maladaptive strategies. Additionally, those in the high loneliness group suppressed emotional expressions and actively rejected or withdrew from others more in comparison to the lower loneliness profiles.

However, since loneliness can also be experienced when one is surrounded by other people (Mansfield et al., 2019), there is an additional need to understand the influence of interpersonal emotion regulation strategies. Interpersonal emotion regulation has been defined in various ways. Some researchers focus on the communicative function of emotional expression and how interaction with others (e.g., their responses) shapes *our own* emotional state (Hofmann, 2014; Zaki & Williams, 2013). An example of this strategy is when we cry and others comfort us, in ways that make us feel better (Sharman et al., 2019). Others have focused on strategies aiming to improve or worsen another person’s emotional state – for example, reminding someone of their previously harmful actions to make them feel guilty, or paying someone a compliment to make them feel proud (Niven, Totterdell, Stride, & Holman, 2011). Robust links between emotional expression and loneliness have been reported in the social cognitive neuroscience literature, as evidenced in increased sensitivity for emotional cues and decreased emotional mimicry in individuals experiencing greater loneliness (Arnold & Winkielman, 2021; Vanhalst, Gibb, & Prinstein, 2017). Links with strategies directed towards others have been relatively underexplored in this domain. Though, given interpersonal emotional regulation strategies are embedded within social contexts, their investigation may provide some insights into linkages with social conceptualizations of loneliness.

### Social identity theory and loneliness

Alongside interpersonal emotion processes, it is also important to explore how more complex aspects of the social context related to loneliness, such as a person’s sense of belonging, group identification, and social support. In this regard, a second theoretical framework that is relevant to these factors is the social identity approach (after Tajfel, Turner, Austin, & Worchel, 1979; Turner, Hogg, Oakes, Reicher, & Wetherell, 1987). This approach argues that people’s sense of self is defined not only by their unique personal characteristics (e.g., as creative, strong, and intelligent) but also by the groups they belong to and identify with (e.g., as members of a particular family, occupational, or recreational group; Jetten, Haslam, & Haslam, 2012). These in‐group memberships and related social identities shape our behaviours and attitudes, creating a sense of shared purpose (Haslam, Jetten, Cruwys, Dingle, & Haslam, 2018). Moreover, social identities promote health and well‐being because when they are a positive source of influence they provide access to key psychological resources including social support, self‐esteem, control, belonging, and meaning (Greenaway, Cruwys, Haslam, & Jetten, 2016; Haslam et al., 2008; Jetten et al., 2012, 2015; Steffens, Jetten, Haslam, Cruwys, & Haslam, 2016).

As a corollary of this analysis, the social identity approach views loneliness as resulting partly from the loss or lack of important social group memberships and associated social identities. Here, the painful emotional experience of loneliness is seen to result not only from the lack or loss of group‐based social connections but also from the associated loss of access to key psychological resources (Haslam et al., 2022). Seeking to address this, interventions that focus on maintaining and increasing group memberships and identifications have been successful not only in increasing these psychological resources but also in reducing social anxiety and loneliness (Cruwys et al., 2021; Haslam, Cruwys, et al., 2019). Consequently, this approach suggests that interventions that focus on developing and maintaining positive group memberships and identities can be an important way to address loneliness, and ultimately, to improve mental health (Dingle et al., 2021).

Group processes have also been shown to have an important role to play in the experience of loneliness (Wakefield, Bowe, Kellezi, Mcnamara, & Stevenson, 2019). For instance, the number of groups in an individual’s social network has been shown to significantly improve adjustment, loneliness, and various other indicators of mental health (Cruwys et al., 2013; Lam et al., 2018). However, group membership alone is not sufficient to ensure these outcomes. Instead, like health and well‐being in general, loneliness is understood to be shaped by a person’s sense of identification with relevant groups (Haslam et al., 2018; Jetten et al., 2012; Sani, Herrera, Wakefield, Boroch, & Gulyas, 2012). Therefore, in this context, one of the most important resources is social support (Guan & So, 2016; Junker, Dick, Avanzi, Häusser, & Mojzisch, 2019).

Nevertheless, evidence of links between social identification and loneliness is limited and these have typically been explored in the context of intervention studies (Haslam, Cruwys, et al., 2019; Kellezi et al., 2019). Other studies have rarely focused explicitly on loneliness but instead have examined its role in mediating the relationship between social group identification and health (McIntyre, Worsley, Corcoran, Harrison Woods, & Bentall, 2018; Wakefield et al., 2019).

### The intersection between social identity and emotion regulation

While currently unexplored in the loneliness literature, links between social identity and emotion regulation frameworks have been a focus for studies of well‐being and adjustment in disadvantaged adults with mental health problems. Here, there has been a tendency to prioritize one dimension over the other. The *emotion‐primary model* is one favoured by clinical psychologists. In this model, adaptive emotion regulation is considered to be a starting point from which an individual can develop better relationships and social connections (Dingle, Brander, Ballantyne, & Baker, 2013). The *social‐primary model* is favoured by social psychologists (Haslam, Haslam, Jetten, Cruwys, & Bentley, 2019b). This model suggests that groups and social identities are the foundation from which people can access a range of psychological resources. While social identity research has not explored emotion regulation as a specific psychological resource afforded by group membership, there is evidence that self‐regulation was a vehicle through which group belonging reduced depression in people with acquired brain injury (Kinsella, Muldoon, Fortune, & Haslam, 2020).

While there is growing evidence for both models, researchers have also found evidence of a bidirectional relationship. For instance, Walter (2017) who examined the links between emotion regulation, social support, and well‐being in residents of a homeless accommodation service over the course of a year, found support for both models. The finding that those with worse emotion regulation experienced less social support and lower well‐being supported the emotion‐primary model. And evidence showing that those with better social support were better able to regulate their negative emotions and experienced better well‐being supported the social‐primary model. This focus on the dual influences of emotion regulation and social factors has clearly enhanced our understanding of mental health and well‐being in this population. The focus of the present research is whether an integrated social‐emotional model can provide us with a better understanding of loneliness experiences.

### The present study

Drawing on the above research, the aim of the present study was to investigate relationships between emotion regulation, social identity, and loneliness among people with and without a history of mental illness (MH Hx vs. No MH Hx). The study also examined the inter‐relationships between these variables to better understand their unique and combined contributions to the loneliness of each group, which was tested in a series of emotion‐primary and social‐primary hierarchical regression analyses. Emotion regulation was measured along with both the intrinsic/extrinsic (i.e., internal/interpersonal) and affect improving/worsening dimensions to fully capture the range of strategies in use. Social identity was measured using the number of groups, the strength of connectedness with multiple groups, and social support both given and received by respondents. Demographic factors were controlled for in the context of comparing the contributions to loneliness variance from both an emotion‐primary model (in which emotion regulation factors were entered first and social identity factors second) and a social‐primary model (in social identity factors were entered first and emotion regulation factors second).

Drawing on previous clinical research (Dingle, Williams, Jetten, & Welch, 2017; Eres et al., 2020), it was expected that the MH Hx group would report more emotion regulation problems and be more socially disconnected than the No MH Hx groups. It was hypothesized that this would be reflected in lower scores on measures of social factors (fewer groups, a lower sense of connectedness to multiple groups, and less perceived support) and use of affect improving strategies (both internal and interpersonal; H1a), paired with higher scores on measures of affect worsening strategies (both internal and interpersonal) and loneliness (H1b). Based on previous investigations of social identity and emotion regulation contributions to well‐being (Walter, 2017), it was also hypothesized that there would be an association between loneliness and indicators of emotion‐ and social‐primary frameworks in both groups (H2). This would be indicated if both the emotion‐ and social‐primary approaches accounted for significant amounts of variance in loneliness as tested in a hierarchical regression.

## Method

### Participants

This study recruited participants online using Prolific, a dedicated platform for online subject recruitment that addresses many of the concerns associated with other online sampling platforms (e.g., related to identity verification and selective attrition; Palan & Schitter, 2018). However, there are also risks and limitations in using Prolific that are relevant to the present study –in particular, demand effects and sample representativeness. First, research participation on recruitment platforms such as MTurk is typically influenced by compensation rates, particularly as a function of task length (Buhrmester et al., 2011). Prolific reduces this by defining a realistic minimum compensation of £5 per hour of task (Palan & Schitter, 2018). The protocol that this study was embedded in aimed to reduce the likelihood of demand effects further by limiting the length of the survey task and not offering greater incentivization by compensating beyond the minimum rate. Second, sample representativeness of recruitment platforms has been found to be more representative than convenience samples such as university students, but less representative than national probability samples (Berinsky, Huber, & Lenz, 2012). This protocol addressed this by utilizing nationally representative sampling of the United Kingdom (UK) population through Prolific to maximize the generalisability of the data and findings. This representative sample was stratified across three main demographics (age, gender, and ethnicity) based on data from the UK Office of National Statistics. The sample was divided into subgroups with the same proportions as the national population (e.g., the proportion of 28‐ to 37‐year‐old Asian males; Prolific Team, 2021). Data collection occurred between the 9^th^ and 11^th^ of June 2020 during the first phase of the coronavirus (COVID‐19) pandemic. As a result, two single‐item measures were added to assess the overall perceived personal impact of the pandemic on the participant and the level of physical distancing as a function of current self‐isolation, work, and travel.

A total of 1,036 participants aged 18–82 years were initially recruited from the online survey platform Prolific, of which 1,005 completed the survey. Following data cleaning and assumption checking, 875 participants remained. Of these, 658 (75.2%) indicated that they had no current or previous mental health diagnosis, and were allocated to the No MH Hx group, while the remaining 217 (24.8%) indicated having a current or previous mental health diagnosis and thus were allocated to the MH Hx group. Demographic characteristics for both groups are reported in Table 1. Demographic characteristics for both groups are reported in Table 1. Examination of these sub‐samples revealed the MH Hx group comprised significantly more people identifying as female and nonbinary than male compared to the No MH Hx group, χ^2^ (2, *N* = 875) = 11.47, *p* = .003. The MH Hx group also reported a greater perceived personal impact of COVID‐19 on their life than the No MH Hx group, *F*(1,873) = 15.55, *p* < .001. No other differences emerged. To account for these significant differences, demographic and COVID‐19 items were controlled for in the partial correlations and hierarchical regressions.

**Table 1:** Demographic characteristics of the participant sample for those with and without a history of mental illness

Variable	No MH Hx (*N* = 658)					MH Hx (*N* = 217)				
	*N*	%	M	*SD*	Range	*N*	%	M	*SD*	Range
Age			46.36	15.67	18–82			44.77	13.86	20–75
Gender										
Male	338	51.52				87	40.28			
Female	318	48.48				128	59.26			
Other	0	0				1	0.46			
Relationship status										
Single	110	16.77				56	25.93			
In a relationship	64	9.76				21	9.72			
Married/domestic partnership	426	64.94				119	55.09			
SeparateddDivorced	42	6.4				15	6.94			
Widowed	13	1.98				5	2.31			
Education level										
Year 10 or below	7	1.07				2	0.93			
Year 11–12	96	14.63				31	14.35			
Vocational college/TAFE	157	23.93				49	22.69			
Bachelor’s degree	270	41.16				85	39.35			
Postgraduate degree	126	19.21				49	22.69			
COVID‐19 impact			2.73	1.01	1–5			2.96	1.06	1–5
Physical distancing										
Self‐isolation: infection/close contact	85	12.96				32	14.82			
Self‐Isolation: vulnerability to COVID‐19										
At‐home work, necessary travel/activities only	408	62.2				126	58.33			
Regular work, necessary travel/activities only	69	10.52				20	9.26			
Regular work, travel, and activities	17	2.59				10	4.63			
Other	76	11.59				28	12.96			

### Measures

#### Group listing

Adapted from the Haslam et al. (2008) measure, participants were asked to identify up to five groups they belonged to. Participants who were unable to list any groups were provided with the option to skip the remaining social measures and continued straight to the items on loneliness. The listing for this study was capped at 5 groups due to constraints associated with being embedded in a larger survey protocol.

#### Multiple group memberships

The strength of connectedness with multiple groups was assessed using the Multiple Group Membership Scale; a 4‐item measure that has been used with a range of groups and studies (Haslam et al., 2008; Jetten et al., 2015; Jetten, Haslam, Pugliese, Tonks, & Haslam, 2010). Higher scores indicated a greater sense of belonging to multiple groups. All items were scored on a scale from 1 (do not agree at all) to 7 (agree completely). Internal consistencies were α = 0.93 and 0.92 for the No MH Hx and MH Hx subsamples, respectively.

#### Social support given and received

Social support given and received was measured using two 4‐item scales used extensively in social identity research (Steffens et al., 2016). All items were scored on a scale from 1 (not at all) to 7 (definitely). Internal consistencies of the given and received were α = 0.89 and 0.92 for the No MH Hx subsample, and α = 0.88 and 0.92 for the MH Hx subsample.

#### Emotion regulation of others and self (EROS)

Emotion regulation was measured using the EROS (Niven et al., 2011), a 19‐item measure of internal and interpersonal regulation and dysregulation. The measure comprised four subscales: extrinsic affect improving (6‐items), extrinsic affect worsening (3‐items), intrinsic affect improving (6‐items), and intrinsic affect worsening (4‐items). All items were scored on a scale of 1 (not at all) to 5 (a great deal). An average score was calculated for each subscale, where higher scores indicated more frequent use of that category of emotion regulation strategy. The internal consistencies of the four subscales (extrinsic affect improving α = 0.89, 0.91; extrinsic affect worsening α = 0.75, 0.7; intrinsic affect improving α = 0.87 0.89; and intrinsic affect worsening α = 0.89, 0.9) were adequate‐to‐good for both the No MH Hx and MH Hx groups, respectively.

#### 8‐item roberts UCLA loneliness scale (RULS‐8)

Loneliness was measured using the RULS‐8 (Roberts, Lewinsohn, & Seeley, 1993). Two of the items are reverse scored (‘*I am an outgoing person’* and *‘I can find companionship when I want’*). Participants responded to each item on a scale of 1 (never) to 4 (always). A total loneliness score was then calculated, with a possible range of 8–32 and where higher values indicated greater perceived loneliness. Internal consistencies for the scale were α = 0.88 and 0.87 for the No MH Hx and MH Hx subsamples, respectively.

#### Demographics and COVID‐19

Participants were asked to indicate their age, gender, relationship status, level of education, and mental health history. As the COVID‐19 pandemic was unfolding at the time the study was conducted, two items were included to attempt to control for its influence. The overall perceived personal negative impact of the COVID‐19 pandemic was assessed by a single 5‐point item from 1 (not at all) to 5 (extremely). Participants also indicated what level of physical distancing they were experiencing at the time of the survey by selecting the option that described their situation the best regarding self‐isolation or regular/adjusted work, travel, and activities.

The measures in this study were part of a larger survey used to examine social and emotional factors in loneliness, mental health, and well‐being. The full survey comprised 21 measures of various clinically and socially relevant variables.

### Procedure

Eligible participants were notified about the study on the Prolific platform where information about the study title, a brief description, and the amount of compensation received for completion were provided. Upon clicking on the link to the study, participants were routed to an information page that provided details of the study and a consent form. Consenting participants then completed the survey on a personal digital device at their convenience. Upon completion, participants were routed to a debrief sheet and a link to return to Prolific and submit their completion code for reimbursement. The whole survey took approximately 15 min to complete, and participants were compensated £1.25 upon completion.

### Analytic strategy

Missing data analyses and assumption checks were conducted on the dataset (archived on Mendeley Data; Hayes, 2022) using SPSS, Version 27. A one‐way MANOVA was used to determine group differences (MH, No MH Hx) in social and emotional variables. Partial correlation and regression analyses were then performed using Jamovi for each group separately. In the partial correlations, we controlled for demographic (age, gender, relationship status, level of education, and mental health history) and COVID‐related factors (perceived impact of COVID‐19 and current level of physical distancing) to avoid spurious correlations between the key variables that may be driven by these factors. Additionally, a Bonferroni adjustment was applied to the partial correlations using the eight social identity and emotion regulation variables to reduce potential problems associated with multiplicity. In the hierarchical regression, demographics and COVID‐19 items were entered into the first step of all models as control variables. In the emotion‐primary model, emotion regulation variables were entered in the second step followed by variables assessing social factors in the third step. In the social‐primary model, social factors were entered in the second step followed by emotion regulation in the third step. We assessed the amount of variance that each model explained at each step (*R* ^2^) and model fit as indicated by estimated error (RMSE) for both the MH Hx and No MH Hx groups, while individual variables were examined using standardized coefficients.

## Results

### Preliminary analyses

Prior to running the main analyses, missing data analysis and assumption checking were performed. Of the 1044 responses collected, 162 did not complete the survey properly and were deleted. Following this, a further 7 multivariate outliers were found and deleted, leaving 875 responses for analyses. After checking assumptions of normality, several variables were found to have significant positive skew: age, physical distancing, extrinsic affect worsening strategies, and intrinsic affect worsening strategies. These were transformed appropriately to the severity of skew (square‐root for mild, logarithmic for moderate, and inverse for severely skewed data). Assumptions of homogeneity of variance–covariance (Box’s *M* = 55.89, *p* = .146), homoscedasticity, independence of observations (Durbin–Watson = 1.89–2.04), linearity, multicollinearity (VIFs ≤ 1.47, tolerances ≥ .64), and normality of residuals were met for both groups.

### Hypothesis 1: Descriptives and group differences

There was a significant overall difference in the variables examined between the No MH Hx and MH Hx groups, *F*(9, 865) = 7.51, Wilk’s Λ = 0.93, *p* < .001, η^2^ = 0.07. Consistent with H1, the MH Hx group had significantly higher intrinsic affect worsening strategies and loneliness than the No MH Hx group. Unique effect sizes for both these measures were small (η_p_^2^ = .04 and .06, respectively). However, there were no differences between groups on the remaining variables, so there was only partial support for H1 (see Table 2).

**Table 2:** Descriptives and between‐groups effects of key variables across MH Hx and No MH Hx groups

Measure	No MH Hx		MH Hx		*F*(1,873)	*p*	η_p_^2^
	*M*	*SD*	*M*	*SD*			
Number of groups	3.81	1.3	3.79	1.3	0.02	.888	0
Multiple group membership	3.58	1.53	3.38	1.62	2.64	.104	0
Social support given	5.59	1.05	5.65	1.07	0.49	.485	0
Social support received	5	1.3	4.82	1.35	3.21	.073	0
Extrinsic affect improving strategies	3.56	0.86	3.67	0.92	2.83	.093	0
Extrinsic affect worsening strategies	1.4	0.61	1.39	0.58	0.22	.642	0
Intrinsic affect improving strategies	3.13	0.86	3.02	0.92	2.89	.089	0
Intrinsic affect worsening strategies	1.6	0.76	1.99	0.98	35	<.001	.04
Loneliness	18.65	3.56	20.43	3.47	41.31	<.001	.06

### Hypothesis 2: Correlations and hierarchical regressions models

Consistent with H2, loneliness was found to be significantly correlated with six of the eight variables in the No MH Hx group when controlling for demographic information and applying a Bonferroni adjustment (α_ajusted_ = .006). Specifically, loneliness was positively associated with intrinsic affect worsening strategy use (*p* < .001) and negatively associated with multiple group memberships (*p* < .001), giving social support (*p* < .001), receiving social support (*p* < .001), and intrinsic affect improving strategy use (*p* = .003). Unexpectedly, extrinsic affect worsening strategy use was also negatively associated with loneliness (*p* < .001). These variables had a range of significant interrelationships with each other (absolute *r* = .11, *p* = .004 to *r* = .57, *p* < .001; Table 3).

**Table 3:** Partial correlations between key variables when controlling for demographic information with a Bonferroni adjustment. Correlations below the diagonal are for the No MH Hx group, while correlations above the diagonal are for the MH Hx group

Variable	1	2	3	4	5	6	7	8	9
1. Number of groups listed	1	0.37^**^	0.09	0.12	0.15	−0.06	0.18	−0.04	0.02
2. Multiple group membership	0.37^**^	1	0.29^**^	0.40^**^	0.26^**^	−0.10	0.39^**^	−0.11	−0.09
3. Social support given	0.04	0.27^**^	1	0.50^**^	0.59^**^	−0.11	0.43^**^	−0.01	−0.10
4. Social support received	0.11^*^	0.38^**^	0.57^**^	1	0.28^**^	0.05	0.43^**^	−0.14	−0.35^**^
5. Extrinsic affect improving	0.12^*^	0.27^**^	0.49^**^	0.28^**^	1	−0.20^*^	0.57^**^	−0.02	−0.10
6. Extrinsic affect worsening	−0.1	0.02	0.12	0.08	−0.09	1	−0.14	−0.23^**^	−0.04
7. Intrinsic affect improving	0.13^**^	0.32^**^	0.31^**^	0.34^**^	0.54^**^	−0.13^**^	1	−0.13	−0.25^**^
8. Intrinsic affect worsening	0.00	−0.08	−0.18^**^	−0.18^**^	0.04	−0.37^**^	−0.02	1	0.36^**^
9. Loneliness	−0.05	−0.18^**^	−0.15^**^	−0.39^**^	−0.07	−0.14^**^	−0.12^*^	0.39^**^	1

*α_adjusted_ = .006, ^**^ α_adjusted_ ≤ .001.

Control variables: age, gender, relationship status, level of education, perceived COVID impact, and physical distancing.

In the MH Hx group, loneliness was significantly correlated with only one social identity variable and two emotion regulation strategies when controlling for demographic information. Specifically, loneliness was associated with intrinsic affect worsening strategy use (*p* < .001) and negatively associated with receiving social support (*p* < .001) and use of intrinsic affect improving strategies (*p* < .001). Surprisingly, the number of groups, multiple group memberships, social support received, and use of extrinsic affect worsening strategies were not associated with loneliness. The variables also had multiple significant interrelationships with each other (absolute *r* = .2, *p* = .003 to *r* = .59, *p* < .001; Table 3).

Consistent with H2, regression analyses testing both the social‐ and emotion‐primary models explained 37% of the variance in loneliness within the No MH Hx group. Specifically, each step of the reciprocal models explained the same amount of variance (*R* ^2^), change in variance explained (Δ*R* ^2^), and had the same estimation of error (RMSE) (Table 4). Regarding individual variables, being male with reference to being female (β = −0.16, *p* = .015), being married/in a domestic partnership with reference to being single (β = −0.34, *p* < .001), COVID‐19 impact (β = 0.15, *p* < .001), social support given (β = 0.16, *p* < .001), social support received (β = −0.38, *p* < .001), and intrinsic affect worsening strategy use (β = 0.38, *p* < .001) were significant in the final step of both the emotion‐ and social‐primary models. Age (β = −0.17, *p* < .001) and being in a relationship (not cohabitating) with reference to being single (β = −0.28, *p* = .044) were significant in Step 2 of the social‐primary approach (β = −0.17, *p* < .001) but were non‐significant in Step 3. Intrinsic affect improving strategy use was significant in Step 2 (β = −0.09, *p* = .026) of the emotion‐primary approach but was non‐significant in Step 3 (refer to Tables A1 and Table B1 in the Appendix).

**Table 4:** Summaries for the emotion‐ and social‐primary regression models of loneliness

Step	Participants with No mental health history								Participants with a mental health history							
	Emotion‐primary				Social‐primary				Emotion‐primary				Social‐primary			
	*R* ^2^	Δ*R* ^2^	RMSE	*p*	*R* ^2^	Δ*R* ^2^	RMSE	*p*	*R* ^2^	Δ*R* ^2^	RMSE	*p*	*R* ^2^	Δ*R* ^2^	RMSE	*p*
1	.13		3.33	<.001	.13		3.33	<.001	.14		3.21	.019	.14		3.21	.019
2	.27	.14^***^	3.04	<.001	.27	.14^***^	3.05	<.001	.29	.15^***^	2.92	<.001	.25	.11^***^	3	<.001
3	.37	.1^***^	2.83	<.001	.37	.1^***^	2.83	<.001	.35	.06^***^	2.79	<.001	.35	.11^***^	2.79	<.001

Emotion‐primary: demographics (Step 1), emotion regulation (Step 2), and social identity factors (Step 3).

Social‐primary: demographics (Step 1), social identity factors (Step 2), and emotion regulation (Step 3).

Δ*R* ^2^ Sig: ^***^ *p* ≤ .001.

Similarly, regression analyses testing both the emotion‐ and social‐primary models explained 35% of the variance in loneliness in the MH Hx group. Here there was a different pattern of results. While Steps 1 and 3 of both models explained the same amount of variance and had the same error estimation, Step 2 of the emotion‐primary model explained more variance (15%) and had a smaller error estimation than Step 2 of the social‐primary model (11% of the variance in loneliness; see Table 4). Regarding individual variables, social support received (β = −0.32, *p* < .001), intrinsic affect improving (β = −0.16, *p* = .047), and intrinsic affect worsening (β = 0.33, *p* < .001) strategy use were significant in the final step of both models. COVID‐19 impact (β = 0.13, *p* = .045) was significant in Step 2 of the emotion‐primary model and non‐significant in Step 3. Age (β = −0.15, *p* = .034) and COVID‐19 impact (β = 0.13, *p* = .047) were significant in Step 2 of the social‐primary approach and non‐significant in Step 3 (refer to Table B2, B3 and B4 in the Appendix).

## Discussion

The purpose of the present study was to examine the unique and combined contributions of emotion regulation strategies and social group connections and support to loneliness in individuals with and without a history of mental health problems. In line with H1, respondents with a mental illness history reported greater use of internal affect worsening strategies (such as rumination) and greater perceived loneliness than those without such a history. These results are consistent with previous work suggesting that affect worsening emotion regulation strategies might be a transdiagnostic factor for mental illnesses such as depression and anxiety (Hofmann et al., 2012), posttraumatic stress disorder (Tull, Barrett, McMillan, & Roemer, 2007), substance use disorders (Dingle et al., 2018), and personality disorders (Gratz, Rosenthal, Tull, Lejuez, & Gunderson, 2006). Furthermore, increasing emotion regulation capacity is a predictor of therapeutic improvement (Bradley et al., 2011; Hofmann et al., 2012). However, contrary to expectation, we found no significant differences between groups on affect improving emotion regulation strategies or social indicators. In the context of previous research, this suggests that the presence of maladaptive emotion regulation strategies – as opposed to adaptive alternatives – may be a primary factor in the development and continuation of mental illness. Our results also extend the notion of transdiagnostic risk factors to include loneliness, which was indeed higher in those with a history of mental illness than in those without such a history (Wilkialis et al., 2021).

Full support was found for H2 in both subsamples. Emotion regulation explained significant amounts of unique variance in reported loneliness when controlling for demographic factors and social identity variables, and alternatively, social identity variables explained unique variance when controlling for demographic factors and emotion regulation. These analyses indicated that social support received from groups and use of internal affect worsening strategies were significantly associated with loneliness for both those with and without a history of mental illness. These findings are consistent with previous studies which have found that negative reappraisal has a strong relationship with the painful emotional experience of loneliness (Kearns & Creaven, 2017; Preece et al., 2021). Likewise, social support received was also significantly associated with loneliness in both regression models in both subsamples, thereby corroborating previous evidence that perceived social support is an important factor in attenuating experiences of loneliness (Solomon, Bensimon, Greene, Horesh, & Ein‐Dor, 2015; Van Den Brink et al., 2018).

Examination of the two groups separately also revealed other significant associations. Among those without a history of mental illness, there was a significant association between loneliness and social support given, with internal affect improving strategies becoming non‐significant following the inclusion of social identity variables. In contrast, among those with a history of mental illness, there was a significant association between loneliness and intrinsic affect improving strategies. While the remaining social identity variables (i.e., multiple group membership and social support given) and interpersonal emotion regulation strategies were not significant predictors of loneliness for those with a history of mental illness, they were significantly correlated with loneliness and with each other. This suggests that emotion regulation processes may play a more influential role in loneliness for those with a mental illness history.

Taken together, the results of these analyses suggest the social and emotional elements of loneliness examined in the present study have a complex relationship both with each other and with the broader construct of loneliness in general. In particular, the findings build upon the pre‐existing social needs and cognitive discrepancy models by going beyond an analysis of actual and perceived social interaction to demonstrate the relevance of social support and intrinsic emotion regulation strategies to our understanding of loneliness. The findings also support previous research highlighting the importance of positive social identification in improving maladaptive cognitive structures such as social isolation schema (Cruwys et al., 2014). Furthermore, while the cross‐sectional nature of the present study limits the causal inferences we can draw in relation to social support and internal emotion regulation strategies, it is possible that these two processes contribute to change in perceptions of loneliness, and in turn mental health (Wang, Mann, Lloyd‐Evans, Ma, & Johnson, 2018). However, the notion that interpersonal emotion regulation strategies play a role in loneliness due to their social context and conceptual links with social identity was not supported by the results of the regression models.

These results also have practical implications for the management of loneliness in clinical and community settings. First, while loneliness is not a recognized clinical condition, it is nevertheless important to screen for people’s perceptions of loneliness alongside social group connectedness and maladaptive emotion regulation during intake as this may provide clinicians with an early indication of their vulnerability and risk of developing psychological distress or disorder symptomology (Macneil, Hasty, Conus, & Berk, 2012). Second, identifying potential problems with loneliness, maladaptive emotion regulation, and social support helps clinicians to identify when it might be important to provide education about the role of these factors in enhancing mental health (Lyman et al., 2014). Finally, assessing these influential factors would allow clinicians to target them for principal or auxiliary intervention by drawing on the most appropriate of the range of available responses – from individual cognitive‐behavioural therapy to identity‐based group interventions (Haslam, Cruwys, et al., 2019; Käll, Backlund, Shafran, & Andersson, 2020).

### Limitations and future research

This study was limited by the use of cross‐sectional data, as this precludes causal and temporal inferences about the directional and dual effects of the hypothesized processes on loneliness. Future studies should therefore aim to build on the present research by fleshing out an integrated socio‐emotional model of loneliness and testing this in studies that use longitudinal methods to reduce intra‐individual variance and establish the causal impact of these factors (Ployhart & MacKenzie, 2015). Additionally, it is important to consider the limited scope we have for interpreting the difference in internal affect‐worsening strategies given the emotion regulation measure that we used. In particular, the internal worsening subscale was not developed to index the many specific dimensions of maladaptive emotion regulation. Rather, it is limited to negative reappraisal strategies, akin to rumination, and does not capture other strategies such as avoidance or suppression that are also linked with loneliness (Niven et al., 2011; Preece et al., 2021). Future research that measures these additional dimensions and strategies, will help to provide a more complete picture of the role of the multiple aspects of emotion regulation that might affect loneliness.

Finally, the study was undertaken during the initial period of the COVID‐19 pandemic when strict levels of physical distancing and the transition to virtual spaces were introduced. However, despite this transition, those without a history of mental illness reported levels of loneliness comparable to those observed in pre‐pandemic studies (Hudiyana et al., 2021; Wu & Yao, 2008). Although we collected data on responses to the pandemic and controlled for these in the analyses, it is possible that these social adjustments may in turn have limited people’s ability to help or hinder others through interpersonal interactions even though loneliness remained relatively stable (Muldoon, 2020). Interpersonal emotion regulation opportunities, and in turn the related internal emotion regulation and social group processes, may have been influenced as a result.

Strengths of the current research include the utilization of a large sample representative of the UK population, allowing the observed results to be more generalizable to similar populations. Another strength was that we combined the current and previous mental illness diagnosis groups, which serves to increase confidence in the generalisability and practical relevance for both mental health clients and practitioners (Murad, Katabi, Benkhadra, & Montori, 2018).

### Conclusion

This study is one of the first to examine loneliness by focusing on social and emotional processes – rather than social and emotional subtypes of loneliness – and to do so in the context of differences in mental health. We found that social identity and emotion regulation both account for significant variation in reported loneliness both when controlling for each other and in combination. Overall, the study provides early evidence that emotional and social processes operate together to shape experiences of loneliness – suggesting that the negative emotional experience of loneliness is shaped by a person’s social context, and that a person’s social context can elicit feelings of loneliness if their groups and relationships are emotionally triggering and not supportive. These insights have important implications for theory and practice that we hope future research will continue to build on.

## Author contributions

**Catherine Haslam** (Writing – review & editing) **Genevieve Dingle** (Conceptualization; Funding acquisition; Supervision; Writing – review & editing) **S. Alex Haslam** (Conceptualization; Writing – review & editing) **Shaun Hayes** (Conceptualization; Data curation; Formal analysis; Investigation; Methodology; Writing – original draft; Writing – review & editing) **Molly Carlyle** (Writing – review & editing).

## Conflicts of interest

The authors have no conflicts of interest to declare.

## Data Availability

The data that support the findings of this study will be made openly available on Mendeley Data at https://doi.org/10.17632/nkzwnj24zy.1.

## Appendix 1 MH Hx demographic and assumption checks

**Table A1:** Current vs. previous MH Dx

Variable	Previous Dx (*N* = 117)					Current Dx (*N* = 100)				
	*N*	%	*M*	*SD*	Range	*N*	%	*M*	*SD*	Range
Age			46.8	13.1	23–75			42.16	14.34	20–67
Gender										
Male	41	35				46	46			
Female	76	65				53	53			
Other						1	1			
Relationship status										
Single	30	26				25	25			
In a relationship	10	9				11	11			
Married/domestic partnership	68	58				53	53			
Separated/divorced	6	5				9	9			
Widowed	3	3				2	2			
Education level										
Year 10 or below	1	1				1	1			
Year 11–12	17	15				14	14			
Vocational college/TAFE	21	18				28	28			
Bachelor’s degree	50	43				36	36			
Postgraduate degree	28	24				21	21			
COVID‐19 impact			3.03	0.995	1–5			2.9	1.115	1–5
Physical distancing										
Self‐isolation: infection/close contact	1	1%				1	1%			
Self‐isolation: vulnerability to COVID‐19	17	15%				13	13%			
At‐home work, necessary travel/activities only	69	59%				59	59%			
Regular work, necessary travel/activities only	8	7%				11	11%			
Regular work, travel, and activities	5	4%				5	5%			
Other	17	15%				11	11%			

## Appendix B Regression models

**Table B1:** No MH Hx emotion‐primary regression model

Variable	*B*	*SE*(*B*)	β	*p*
Step 1				
Age (Log)	−2.98	0.99	−0.14	.003
Gender				
Male–female	−0.44	0.27	−0.12	.104
Relationship status				
In a relationship (not cohabitating) – single	−0.85	0.54	−0.24	.115
Married/domestic partnership – single	−1.19	0.39	−0.33	.002
Separated/divorced – single	−0.48	0.65	−0.14	.457
Widowed – single	−0.57	1.03	−0.16	.580
Education level				
Year 11–12 – Year 10 or below	−3.18	1.34	−0.89	.018
Vocational college/TAFE – Year 10 or below	−2.49	1.32	−0.70	.061
Bachelor's degree – Year 10 or below	−3.22	1.32	−0.90	.015
Postgraduate degree – Year 10 or below	−3.25	1.34	−0.91	.015
Covid‐19 impact	0.86	0.14	0.24	<.001
Physical distancing				
Self‐isolation: infection/close contact – regular work, travel, and activities	−1.22	1.89	−0.34	.518
Self‐isolation: vulnerability to COVID‐19 – regular work, travel, and activities	0.10	0.92	0.03	.912
At‐home work, necessary travel/activities only – regular work, travel, and activities	0.25	0.85	0.07	.765
Regular work, necessary travel/activities only – regular work, travel, and activities	0.38	0.92	0.11	.677
Other – regular work, travel, and activities	−0.23	0.92	−0.07	.800
Step 2				
Age (Log10)	0.83	1.03	0.04	.421
Gender				
Male–female	−0.48	0.25	−0.13	.059
Relationship status				
In a relationship (not cohabitating) – single	−1.00	0.50	−0.28	.044
Married/domestic partnership – single	−1.31	0.36	−0.37	<.001
Separated/divorced – single	−0.38	0.60	−0.11	.523
Widowed – single	−0.99	0.95	−0.28	.302
Education level				
Year 11–12 – Year 10 or below	−1.78	1.24	−0.50	.152
Vocational college/TAFE – Year 10 or below	−1.08	1.22	−0.30	.377
Bachelor's degree – Year 10 or below	−1.86	1.22	−0.52	.128
Postgraduate degree – Year 10 or below	−1.87	1.24	−0.53	.130
Covid‐19 impact	0.60	0.13	0.17	<.001
Physical distancing				
Self‐Isolation: infection/close contact – regular work, travel, and activities	0.08	1.74	0.02	.963
Self‐isolation: vulnerability to COVID‐19 – regular work, travel, and activities	0.93	0.85	0.26	.274
At‐home work, necessary travel/activities only – regular work, travel, and activities	0.99	0.78	0.28	.204
Regular work, necessary travel/activities only – regular work, travel, and activities	1.16	0.85	0.32	.174
Other – regular work, travel, and activities	0.68	0.85	0.19	.425
Extrinsic affect improving strategies	−0.11	0.17	−0.03	.519
Extrinsic affect worsening strategies (inverse)	−0.27	0.63	−0.02	.668
Intrinsic affect improving strategies	−0.39	0.17	−0.09	.026
Intrinsic affect worsening strategies (Log10)	8.24	0.84	0.42	<.001
Step 3				
Age (Log10)	−0.00	0.98	−0	.996
Gender				
Male–female	−0.59	0.24	−0.16	.015
Relationship status				
In a relationship (not cohabitating) – single	−0.81	0.47	−0.23	.081
Married/domestic partnership – single	−1.21	0.34	−0.34	<.001
Separated/divorced – single	−0.28	0.56	−0.08	.618
Widowed – single	−0.71	0.90	−0.20	.429
Education level				
Year 11–12 – Year 10 or below	−1.59	1.16	−0.45	.170
Vocational college/TAFE – Year 10 or below	−0.80	1.15	−0.23	.484
Bachelor's degree – Year 10 or below	−1.55	1.14	−0.43	.176
Postgraduate degree – Year 10 or below	−1.65	1.16	−0.46	.156
Covid‐19 impact	0.52	0.12	0.15	<.001
Physical distancing:				
Self‐isolation: infection/close contact – Regular work, travel, and activities	−0.12	1.63	−0.03	.944
Self‐isolation: vulnerability to COVID‐19 – regular work, travel, and activities	0.55	0.80	0.16	.488
At‐home work, necessary travel/activities only – regular work, travel, and activities	0.84	0.73	0.24	.250
Regular work, necessary travel/activities only – regular work, travel, and activities	1.07	0.80	0.30	.178
Other – regular work, travel, and activities	0.72	0.80	0.20	.369
Extrinsic affect improving strategies	−0.18	0.18	−0.04	.322
Extrinsic affect worsening strategies (Inverse)	−0.06	0.60	−0	.913
Intrinsic affect improving strategies	0.02	0.17	0	.915
Intrinsic affect worsening strategies (Log10)	7.44	0.80	0.38	<.001
Number of groups	0.07	0.10	0.03	.446
Multiple group membership	−0.10	0.09	−0.04	.293
Social support given	0.56	0.15	0.16	<.001
Social support received	−1.04	0.11	−0.38	<.001

**Table B2:** No MH Hx social‐primary regression model

Variable	*B*	*SE*(*B*)	β	*p*
Step 1				
Age (log)	−2.98	0.99	−0.14	.003
Gender				
Male–female	−0.44	0.27	−0.12	.104
Relationship status				
In a relationship (not cohabitating) – single	−0.85	0.54	−0.24	.115
Married/domestic partnership – single	−1.19	0.39	−0.33	.002
Separated/divorced – Single	−0.48	0.65	−0.14	.457
Widowed – single	−0.57	1.03	−0.16	.580
Education level				
Year 11–12 – Year 10 or below	−3.18	1.34	−0.89	.018
Vocational college/TAFE – Year 10 or below	−2.49	1.32	−0.70	.061
Bachelor's degree – Year 10 or below	−3.22	1.32	−0.90	.015
Postgraduate degree – Year 10 or below	−3.25	1.34	−0.91	.015
Covid‐19 impact	0.86	0.14	0.24	<.001
Physical distancing				
Self‐Isolation: infection/close contact – regular work, travel, and activities	−1.22	1.89	−0.34	.518
Self‐Isolation: vulnerability to COVID‐19 – regular work, travel, and activities	0.10	0.92	0.03	.912
At‐home work, necessary travel/activities only – regular work, travel, and activities	0.25	0.85	0.07	.765
Regular work, necessary travel/activities only – regular work, travel, and activities	0.38	0.92	0.11	.677
Other – regular work, travel, and activities	−0.23	0.92	−0.07	.800
Step 2				
Age (Log10)	−3.74	0.93	−0.17	<.001
Gender				
Male–female	−0.74	0.25	−0.21	.004
Relationship status				
In a relationship (not cohabitating) – single	−0.69	0.50	−0.19	.165
Married/domestic partnership – single	−1.02	0.36	−0.29	.004
Separated/divorced – single	−0.27	0.60	−0.07	.658
Widowed – Single	−0.20	0.96	−0.06	.834
Education level				
Year 11–12 – Year 10 or below	−2.35	1.24	−0.66	.058
Vocational college/TAFE – Year 10 or below	−1.49	1.23	−0.42	.225
Bachelor's degree – Year 10 or below	−2.23	1.22	−0.63	.068
Postgraduate degree – Year 10 or below	−2.31	1.24	−0.65	.063
Covid‐19 impact	0.77	0.13	0.21	<.001
Physical distancing				
Self‐Isolation: infection/close contact – regular work, travel, and activities	−0.90	1.74	−0.25	.603
Self‐isolation: vulnerability to COVID‐19 – regular work, travel, and activities	0.02	0.85	0.01	.979
At‐home work, necessary travel/activities only – regular work, travel, and activities	0.39	0.78	0.11	.618
Regular work, necessary travel/activities only – regular work, travel, and activities	0.58	0.85	0.16	.499
Other – regular work, travel, and activities	0.14	0.85	0.04	.871
Number of groups	0.08	0.10	0.03	.418
Multiple group membership	−0.12	0.10	−0.05	.208
Social support given	0.38	0.15	0.11	.010
Social support received	−1.14	0.12	−0.42	<.001
Step 3				
Age (Log10)	−0	0.98	−0	.996
Gender				
Male–female	−0.59	0.24	−0.16	.015
Relationship status				
In a relationship (not cohabitating) – single	−0.81	0.47	−0.23	.081
Married/domestic partnership – single	−1.21	0.34	−0.34	<.001
Separated/divorced – single	−0.28	0.56	−0.08	.618
Widowed – single	−0.71	0.90	−0.20	.429
Education level				
Year 11–12 – Year 10 or below	−1.59	1.16	−0.45	.170
Vocational college/TAFE – Year 10 or below	−0.80	1.15	−0.23	.484
Bachelor's degree – Year 10 or below	−1.55	1.14	−0.43	.176
Postgraduate degree – Year 10 or below	−1.65	1.16	−0.46	.156
Covid‐19 impact	0.52	0.12	0.15	<.001
Physical distancing				
Self‐isolation: infection/close contact – regular work, travel, and activities	−0.12	1.63	−0.03	.944
Self‐isolation: vulnerability to COVID‐19 – regular work, travel, and activities	0.55	0.80	0.16	.488
At‐home work, necessary travel/activities only – regular work, travel, and activities	0.84	0.73	0.24	.250
Regular work, necessary travel/activities only – regular work, travel, and activities	1.07	0.80	0.30	.178
Other – regular work, travel, and activities	0.72	0.80	0.20	.369
Number of groups	0.07	0.10	0.03	.446
Multiple group membership	−0.10	0.09	−0.04	.293
Social support given	0.56	0.15	0.16	<.001
Social support received	−1.04	0.11	−0.38	<.001
Extrinsic affect improving strategies	−0.18	0.18	−0.04	.322
Extrinsic affect worsening strategies (inverse)	−0.06	0.60	−0	.913
Intrinsic affect improving strategies	0.02	0.17	0	.915
Intrinsic affect worsening strategies (Log10)	7.44	0.80	0.38	<.001

**Table B3:** MH Hx emotion‐primary regression model

Variable	*B*	*SE*(*B*)	β	*p*
Step 1				
Age (Log)	−2.99	1.84	−0.12	.105
Gender				
Male–female	0.12	0.48	0.03	.809
Other – female	4.21	3.40	1.21	.217
Relationship status				
In a relationship (not cohabitating) – single	−0.16	0.89	−0.05	.854
Married/domestic partnership – single	−0.88	0.61	−0.25	.149
Separated/divorced – single	0.94	1.05	0.27	.370
Widowed – single	2.34	1.66	0.67	.162
Education level				
Year 11–12 – Year 10 or below	−1.04	2.48	−0.30	.675
Vocational college/TAFE – Year 10 or below	−1.45	2.45	−0.42	.555
Bachelor's degree – Year 10 or below	−2.28	2.43	−0.66	.351
Postgraduate degree – Year 10 or below	−1.40	2.45	−0.40	.567
Covid‐19 impact	0.61	0.23	0.18	.008
Physical distancing				
Self‐isolation: infection/close contact – regular work, travel, and activities	−0.41	2.71	−0.12	.879
Self‐isolation: vulnerability to COVID‐19 – regular work, travel, and activities	0.17	1.25	0.05	.892
At‐home work, necessary travel/activities only – regular work, travel, and activities	0.79	1.11	0.23	.479
Regular work, necessary travel/activities only – regular work, travel, and activities	0.30	1.33	0.09	.821
Other – regular work, travel, and activities	2.01	1.25	0.58	.109
Step 2				
Age (Log10)	0.62	1.87	0.03	.741
Gender				
Male–female	−0.10	0.46	−0.03	.830
Other – female	4.20	3.15	1.21	.184
Relationship status				
In a relationship (not cohabitating) – single	−0.72	0.83	−0.21	.384
Married/domestic partnership – single	−0.99	0.56	−0.28	.081
Separated/divorced – single	0.70	0.97	0.20	.471
Widowed – single	2.61	1.55	0.75	.093
Education level				
Year 11–12 – Year 10 or below	0.02	2.30	0.01	.994
Vocational college/TAFE – Year 10 or below	−0.17	2.27	−0.05	.940
Bachelor's degree – Year 10 or below	−0.82	2.26	−0.24	.719
Postgraduate degree – Year 10 or below	0.01	2.27	0	.995
Covid‐19 impact	0.43	0.21	0.13	.045
Physical distancing				
Self‐isolation: infection/close contact – regular work, travel, and activities	−0.87	2.54	−0.25	.731
Self‐isolation: vulnerability to COVID‐19 – regular work, travel, and activities	0.24	1.14	0.07	.834
At‐home work, necessary travel/activities only – regular work, travel, and activities	0.92	1.03	0.26	.374
Regular work, necessary travel/activities only – regular work, travel, and activities	0.92	1.24	0.26	.460
Other – regular work, travel, and activities	1.61	1.16	0.46	.168
Extrinsic affect improving strategies	0.32	0.31	0.08	.315
Extrinsic affect worsening strategies (inverse)	−0.46	1.09	−0.03	.673
Intrinsic affect improving strategies	−0.88	0.29	−0.23	.003
Intrinsic affect worsening strategies (Log10)	5.81	1.20	0.34	<.001
Step 3				
Age (Log10)	−0.23	1.86	−0.01	.900
Gender				
Male–female	−0.23	0.46	−0.07	.611
Other – female	3.53	3.07	1.02	.251
Relationship status				
In a relationship (not cohabitating) – single	−0.82	0.82	−0.24	.317
Married/domestic partnership – single	−0.98	0.55	−0.28	.076
Separated/divorced – single	0.61	0.94	0.18	.515
Widowed – single	1.91	1.52	0.55	.210
Education level				
Year 11–12 – Year 10 or below	−1.29	2.27	−0.37	.570
Vocational college/TAFE – Year 10 or below	−1.76	2.24	−0.51	.433
Bachelor's degree – Year 10 or below	−2.25	2.23	−0.65	.315
Postgraduate degree – Year 10 or below	−1.42	2.23	−0.41	.525
Covid‐19 impact	0.33	0.21	0.10	.108
Physical distancing				
Self‐isolation: infection/close contact – regular work, travel, and activities	−1.16	2.45	−0.34	.635
Self‐isolation: vulnerability to COVID‐19 – regular work, travel, and activities	0.43	1.11	0.12	.698
At‐home work, necessary travel/activities only – regular work, travel, and activities	0.94	1.00	0.27	.349
Regular work, necessary travel/activities only – regular work, travel, and activities	0.85	1.21	0.25	.480
Other – regular work, travel, and activities	1.17	1.13	0.34	.302
Extrinsic affect improving strategies	0.09	0.34	0.02	.796
Extrinsic affect worsening strategies (inverse)	0.18	1.06	0.01	.869
Intrinsic affect improving strategies	−0.61	0.31	−0.16	.047
Intrinsic affect worsening strategies (Log10)	5.57	1.17	0.33	<.001
Number of groups	0.23	0.17	0.09	.186
Multiple group membership	0.17	0.16	0.08	.285
Social support given	0.41	0.28	0.13	.146
Social support received	−0.83	0.20	−0.32	<.001

**Table B4:** MH Hx social‐primary regression model

Variable	*B*	*SE*(*B*)	β	*p*
Step 1				
Age (Log)	−2.99	1.84	−0.12	.105
Gender				
Male–female	0.12	0.48	0.03	.809
Other–female	4.21	3.40	1.21	.217
Relationship status				
In a relationship (not cohabitating) – single	−0.16	0.89	−0.05	.854
Married/domestic partnership – single	−0.88	0.61	−0.25	.149
Separated/divorced – single	0.94	1.05	0.27	.370
Widowed – single	2.34	1.66	0.67	.162
Education level				
Year 11–12 – Year 10 or below	−1.04	2.48	−0.30	.675
Vocational college/TAFE – Year 10 or below	−1.45	2.45	−0.42	.555
Bachelor's degree – Year 10 or below	−2.28	2.43	−0.66	.351
Postgraduate degree – Year 10 or below	−1.40	2.45	−0.40	.567
Covid‐19 impact	0.61	0.23	0.18	.008
Physical distancing				
Self‐isolation: infection/close contact – regular work, travel, and activities	−0.41	2.71	−0.12	.879
Self‐isolation: vulnerability to COVID‐19 – regular work, travel, and activities	0.17	1.25	0.05	.892
At‐home work, necessary travel/activities only – regular work, travel, and activities	0.79	1.11	0.23	.479
Regular work, necessary travel/activities only – regular work, travel, and activities	0.30	1.33	0.09	.821
Other – regular work, travel, and activities	2.01	1.25	0.58	.109
Step 2				
Age (Log10)	−3.71	1.77	−0.15	.038
Gender				
Male–female	−0.17	0.48	−0.05	.715
Other–female	3.63	3.23	1.05	.262
Relationship status				
In a relationship (not cohabitating) – single	−0.57	0.86	−0.16	.511
Married/domestic partnership – single	−0.94	0.58	−0.27	.106
Separated/divorced – single	0.67	1.00	0.19	.504
Widowed – single	1.23	1.61	0.35	.444
Education level				
Year 11–12 – Year 10 or below	−2.47	2.39	−0.71	.304
Vocational college/TAFE – Year 10 or below	−3.10	2.37	−0.89	.193
Bachelor's degree – Year 10 or below	−3.68	2.35	−1.06	.120
Postgraduate degree – Year 10 or below	−2.86	2.36	−0.82	.228
Covid‐19 impact	0.44	0.22	0.13	.047
Physical distancing				
Self‐isolation: infection/close contact – Regular work, travel, and activities	−0.70	2.58	−0.20	.788
Self‐isolation: vulnerability to COVID‐19 – regular work, travel, and activities	0.50	1.18	0.14	.674
At‐home work, necessary travel/activities only – regular work, travel, and activities	0.96	1.06	0.28	.366
Regular work, necessary travel/activities only – regular work, travel, and activities	0.47	1.26	0.14	.710
Other – regular work, travel, and activities	1.52	1.18	0.44	.200
Number of groups	0.21	0.18	0.08	.249
Multiple group membership	0.06	0.17	0.03	.706
Social support given	0.39	0.25	0.12	.123
Social support received	−1.05	0.21	−0.41	<.001
Step 3				
Age (Log10)	−0.23	1.86	−0.01	.900
Gender				
Male–female	−0.23	0.46	−0.07	.611
Other – female	3.53	3.07	1.02	.251
Relationship status				
In a relationship (not cohabitating) – single	−0.82	0.82	−0.24	.317
Married/domestic partnership – single	−0.98	0.55	−0.28	.076
Separated/divorced – single	0.61	0.94	0.18	.515
Widowed – single	1.91	1.52	0.55	.210
Education level				
Year 11–12 – Year 10 or below	−1.29	2.27	−0.37	.570
Vocational sollege/TAFE – Year 10 or below	−1.76	2.24	−0.51	.433
Bachelor's degree – Year 10 or below	−2.25	2.23	−0.65	.315
Postgraduate degree – Year 10 or below	−1.42	2.23	−0.41	.525
Covid‐19 impact	0.33	0.21	0.10	.108
Physical distancing				
Self‐isolation: infection/close contact – regular work, travel, and activities	−1.16	2.45	−0.34	.635
Self‐isolation: vulnerability to COVID‐19 – regular work, travel, and activities	0.43	1.11	0.12	.698
At‐home work, necessary travel/activities only – regular work, travel, and activities	0.94	1.00	0.27	.349
Regular work, necessary travel/activities only – regular work, travel, and activities	0.85	1.21	0.25	.480
Other – regular work, travel, and activities	1.17	1.13	0.34	.302
Number of groups	0.23	0.17	0.09	.186
Multiple group membership	0.17	0.16	0.08	.285
Social support given	0.41	0.28	0.13	.146
Social support received	−0.83	0.20	−0.32	<.001
Extrinsic affect improving strategies	0.09	0.34	0.02	.796
Extrinsic affect worsening strategies (Inverse)	0.18	1.06	0.01	.869
Intrinsic affect improving strategies	−0.61	0.31	−0.16	.047
Intrinsic affect worsening strategies (Log10)	5.57	1.17	0.33	<.001

## References

[bjc12358-bib-0001] Arnold, A. J. , & Winkielman, P. (2021). Smile (but only deliberately) though your heart is aching: Loneliness is associated with impaired spontaneous smile mimicry. Social Neuroscience, 16(1), 26–38. 10.1080/17470919.2020.1809516 32835612

[bjc12358-bib-0002] Berinsky, A. J. , Huber, G. A. , & Lenz, G. S. (2012). Evaluating online labor markets for experimental research: Amazon.com’s mechanical turk. Political Analysis, 20, 351–368. 10.1093/pan/mpr057

[bjc12358-bib-0003] Berking, M. , & Wupperman, P. (2012). Emotion regulation and mental health. Current Opinion in Psychiatry, 25, 128–134. 10.1097/yco.0b013e3283503669 22262030

[bjc12358-bib-0004] Bradley, B. , Defife, J. A. , Guarnaccia, C. , Phifer, J. , Fani, N. , Ressler, K. J. , & Westen, D. (2011). Emotion dysregulation and negative affect. The Journal of Clinical Psychiatry, 72, 685–691. 10.4088/jcp.10m06409blu 21658350 PMC4605672

[bjc12358-bib-0078] Buhrmester, M. , Kwang, T. , & Gosling, S. D. (2011). Amazon's mechanical turk: A new source of inexpensive, yet high‐quality, data? Perspectives on Psychological Science, 6(1), 3–5. 10.1177/1745691610393980 26162106

[bjc12358-bib-0005] Cruwys, T. , Dingle, G. , Haslam, C. , Haslam, S. A. , Jetten, J. , & Morton, T. A. (2013). Social group memberships protect against future depression, alleviate depression symptoms and prevent depression relapse. Social Science & Medicine, 98, 179–186. 10.1016/j.socscimed.2013.09.013 24331897

[bjc12358-bib-0006] Cruwys, T. , Dingle, G. A. , Hornsey, M. J. , Jetten, J. , Oei, T. P. S. , & Walter, Z. C. (2014). Social isolation schema responds to positive social experiences: Longitudinal evidence from vulnerable populations. British Journal of Clinical Psychology, 53, 265–280. 10.1111/bjc.12042 24417580

[bjc12358-bib-0077] Cruwys, T. , Haslam, C. , Rathbone, J. A. , Williams, E. , Haslam, S. A. , & Walter, Z. C. (2021). Groups 4 Health versus cognitive–behavioural therapy for depression and loneliness in young people: randomised phase 3 non‐inferiority trial with 12‐month follow‐up. The British Journal of Psychiatry, 1–8. 10.1192/bjp.2021.128 35049477

[bjc12358-bib-0007] Dingle, G. , Brander, C. , Ballantyne, J. , & Baker, F. (2013). ‘To be heard’: The social and mental health benefits of choir singing for disadvantaged adults. Psychology of Music, 41, 405–421. 10.1177/0305735611430081

[bjc12358-bib-0008] Dingle, G. , Neves, D. C. , Alhadad, S. S. J. , & Hides, L. (2018). Individual and interpersonal emotion regulation among adults with substance use disorders and matched controls. British Journal of Clinical Psychology, 57, 186–202. 10.1111/bjc.12168 29277906

[bjc12358-bib-0009] Dingle, G. , Sharman, L. , Haslam, C. , Donald, M. , Turner, C. , Partanen, R. , … Van Driel, M. L. (2021). The effects of social group interventions for depression: Systematic review. Journal of Affective Disorders, 281, 67–81. 10.1016/j.jad.2020.11.125 33302192

[bjc12358-bib-0010] Dingle, G. , Williams, E. , Jetten, J. , & Welch, J. (2017). Choir singing and creative writing enhance emotion regulation in adults with chronic mental health conditions. British Journal of Clinical Psychology, 56, 443–457. 10.1111/bjc.12149 28722166

[bjc12358-bib-0011] Ditommaso, E. , & Spinner, B. (1997). Social and emotional loneliness: A re‐examination of weiss’ typology of loneliness. Personality and Individual Differences, 22, 417–427. 10.1016/s0191-8869(96)00204-8

[bjc12358-bib-0012] Dykstra, P. A. , & Fokkema, T. (2007). Social and emotional loneliness among divorced and married men and women: Comparing the deficit and cognitive perspectives. Basic and Applied Social Psychology, 29(1), 1–12. 10.1080/01973530701330843

[bjc12358-bib-0013] Eres, R. , Lim, M. H. , Lanham, S. , Jillard, C. , & Bates, G. (2020). Loneliness and emotion regulation: Implications of having social anxiety disorder. Australian Journal of Psychology, 73(1), 46–56. 10.1111/ajpy.12296

[bjc12358-bib-0014] Gratz, K. L. , Rosenthal, M. Z. , Tull, M. T. , Lejuez, C. W. , & Gunderson, J. G. (2006). An experimental investigation of emotion dysregulation in borderline personality disorder. Journal of Abnormal Psychology, 115, 850–855. 10.1037/0021-843X.115.4.850 17100543

[bjc12358-bib-0015] Green, L. R. , Richardson, D. S. , Lago, T. , & Schatten‐Jones, E. C. (2001). Network correlates of social and emotional loneliness in young and older adults. Personality and Social Psychology Bulletin, 27, 281–288. 10.1177/0146167201273002

[bjc12358-bib-0016] Greenaway, K. H. , Cruwys, T. , Haslam, S. A. , & Jetten, J. (2016). Social identities promote well‐being because they satisfy global psychological needs. European Journal of Social Psychology, 46, 294–307. 10.1002/ejsp.2169

[bjc12358-bib-0017] Guan, M. , & So, J. (2016). Influence of social identity on self‐efficacy beliefs through perceived social support: A social identity theory perspective. Communication Studies, 67, 588–604. 10.1080/10510974.2016.1239645

[bjc12358-bib-0018] Haslam, C. , Cruwys, T. , Chang, M. X. L. , Bentley, S. V. , Haslam, S. A. , Dingle, G. A. , & Jetten, J. (2019). GROUPS 4 HEALTH reduces loneliness and social anxiety in adults with psychological distress: Findings from a randomized controlled trial. Journal of Consulting and Clinical Psychology, 87, 787–801. 10.1037/ccp0000427 31403815

[bjc12358-bib-0019] Haslam, C. , Holme, A. , Haslam, S. A. , Iyer, A. , Jetten, J. , & Williams, W. H. (2008). Maintaining group memberships: Social identity continuity predicts well‐being after stroke. Neuropsychological Rehabilitation, 18(5–6), 671–691. 10.1080/09602010701643449 18924001

[bjc12358-bib-0020] Haslam, C. , Jetten, J. , Cruwys, T. , Dingle, G. , & Haslam, S. A. (2018). The new psychology of health: Unlocking the social cure (1st ed.). New York, NY: Routledge.

[bjc12358-bib-0021] Haslam, S. A. , Haslam, C. , Cruwys, T. , Jetten, J. , Bentley, S. V. , Fong, P. , & Steffens, N. K. (2022). Social identity makes group‐based social connection possible: Implications for loneliness and mental health. Current Opinion in Psychology, 43, 161–165. 10.1016/j.copsyc.2021.07.013 34403958

[bjc12358-bib-0022] Haslam, S. A. , Haslam, C. , Jetten, J. , Cruwys, T. , & Bentley, S. (2019). Group life shapes the psychology and biology of health: The case for a sociopsychobio model. Social and Personality Psychology Compass, 13(8), Article 8. 10.1111/spc3.12490

[bjc12358-bib-0023] Hawkley, L. C. , & Cacioppo, J. T. (2010). Loneliness matters: A theoretical and empirical review of consequences and mechanisms. Annals of Behavioral Medicine, 40, 218–227. 10.1007/s12160-010-9210-8 20652462 PMC3874845

[bjc12358-bib-0076] Hayes, S. (2022). Exploring links between social identity, emotion regulation and loneliness in those with and without a history of mental illness (Version 2) [Dataset]. Mendeley Data. 10.17632/nkzwnj24zy.1 PMC9544806 35141908

[bjc12358-bib-0024] Hofmann, S. G. (2014). Interpersonal emotion regulation model of mood and anxiety disorders. Cognitive Therapy and Research, 38, 483–492. 10.1007/s10608-014-9620-1 25267867 PMC4175723

[bjc12358-bib-0025] Hofmann, S. G. , Carpenter, J. K. , & Curtiss, J. (2016). Interpersonal emotion regulation questionnaire (IERQ): Scale development and psychometric characteristics. Cognitive Therapy and Research, 40, 341–356. 10.1007/s10608-016-9756-2 27182094 PMC4864994

[bjc12358-bib-0026] Hofmann, S. G. , Sawyer, A. T. , Fang, A. , & Asnaani, A. (2012). Emotion dysregulation model of mood and anxiety disorders. Depression and Anxiety, 29, 409–416. 10.1002/da.21888 22430982

[bjc12358-bib-0027] Holt‐Lunstad, J. , Smith, T. B. , Baker, M. , Harris, T. , & Stephenson, D. (2015). Loneliness and social isolation as risk factors for mortality. Perspectives on Psychological Science, 10, 227–237. 10.1177/1745691614568352 25910392

[bjc12358-bib-0028] Hudiyana, J. , Lincoln, T. M. , Hartanto, S. , Shadiqi, M. A. , Milla, M. N. , Muluk, H. , & Jaya, E. S. (2021). How universal is a construct of loneliness? Measurement invariance of the UCLA loneliness scale in Indonesia, Germany, and the United States. Assessment. 10.1177/10731911211034564 34301150

[bjc12358-bib-0029] Ingram, I. , Kelly, P. , Deane, F. P. , Baker, A. L. , Goh, M. C. W. , Raftery, D. K. , & Dingle, G. (2020). Loneliness among people with substance use problems: A narrative systematic review. Drug and Alcohol Review, 39, 447–483. 10.1111/dar.13064 32314504

[bjc12358-bib-0030] Jetten, J. , Branscombe, N. R. , Haslam, S. A. , Haslam, C. , Cruwys, T. , Jones, J. M. , … Zhang, A. (2015). Having a lot of a good thing: Multiple important group memberships as a source of self‐esteem. PLoS One, 10(5), e0124609. 10.1371/journal.pone.0124609 26017554 PMC4446320

[bjc12358-bib-0031] Jetten, J. , Haslam, C. , & Haslam, S. A. (2012). The social cure: Identity, health and well‐being. Hove, East Sussex: Psychology Press.

[bjc12358-bib-0032] Jetten, J. , Haslam, C. , Pugliese, C. , Tonks, J. , & Haslam, S. A. (2010). Declining autobiographical memory and the loss of identity: Effects on well‐being. Journal of Clinical and Experimental Neuropsychology, 32, 408–416. 10.1080/13803390903140603 19787523

[bjc12358-bib-0033] Junker, N. M. , Dick, R. , Avanzi, L. , Häusser, J. A. , & Mojzisch, A. (2019). Exploring the mechanisms underlying the social identity–ill‐health link: Longitudinal and experimental evidence. British Journal of Social Psychology, 58, 991–1007. 10.1111/bjso.12308 30561049

[bjc12358-bib-0034] Käll, A. , Backlund, U. , Shafran, R. , & Andersson, G. (2020). Lonesome no more? A two‐year follow‐up of internet‐administered cognitive behavioral therapy for loneliness. Internet Interventions, 19, 100301. 10.1016/j.invent.2019.100301 32071885 PMC7016261

[bjc12358-bib-0035] Kearns, S. M. , & Creaven, A.‐M. (2017). Individual differences in positive and negative emotion regulation: Which strategies explain variability in loneliness? Personality and Mental Health, 11(1), 64–74. 10.1002/pmh.1363 27766765

[bjc12358-bib-0036] Kellezi, B. , Wakefield, J. R. H. , Stevenson, C. , Mcnamara, N. , Mair, E. , Bowe, M. , … Halder, M. M. (2019). The social cure of social prescribing: A mixed‐methods study on the benefits of social connectedness on quality and effectiveness of care provision. British Medical Journal Open, 9(11), e033137. 10.1136/bmjopen-2019-033137 PMC6887058 31727668

[bjc12358-bib-0037] Kinsella, E. L. , Muldoon, O. T. , Fortune, D. G. , & Haslam, C. (2020). Collective influences on individual functioning: Multiple group memberships, self‐regulation, and depression after acquired brain injury. Neuropsychological Rehabilitation, 30, 1059–1073. 10.1080/09602011.2018.1546194 30457441

[bjc12358-bib-0038] Laddis, A. (2015). The pathogenesis and treatment of emotion dysregulation in borderline personality disorder. The Scientific World Journal, 2015, 179276. 10.1155/2015/179276 26380355 PMC4563094

[bjc12358-bib-0039] Lam, B. C. P. , Haslam, C. , Haslam, S. A. , Steffens, N. K. , Cruwys, T. , Jetten, J. , & Yang, J. (2018). Multiple social groups support adjustment to retirement across cultures. Social Science & Medicine, 208, 200–208. 10.1016/j.socscimed.2018.05.049 29929765

[bjc12358-bib-0040] Lim, M. H. , Eres, R. , & Vasan, S. (2020). Understanding loneliness in the twenty‐first century: An update on correlates, risk factors, and potential solutions. Social Psychiatry and Psychiatric Epidemiology, 55, 793–810. 10.1007/s00127-020-01889-7 32524169

[bjc12358-bib-0041] Lyman, D. R. , Braude, L. , George, P. , Dougherty, R. H. , Daniels, A. S. , Ghose, S. S. , & Delphin‐Rittmon, M. E. (2014). Consumer and family psychoeducation: Assessing the evidence. Psychiatric Services, 65, 416–428. 10.1176/appi.ps.201300266 24445678

[bjc12358-bib-0042] Macneil, C. A. , Hasty, M. K. , Conus, P. , & Berk, M. (2012). Is diagnosis enough to guide interventions in mental health? Using case formulation in clinical practice. BMC Medicine, 10(1), 111. 10.1186/1741-7015-10-111 23016556 PMC3523045

[bjc12358-bib-0043] Mansfield, L. , Daykin, N. , Meads, C. , Tomlinson, A. , Gray, K. , Lane, J. , & Victor, C. (2019). A conceptual review of loneliness across the adult life course (16+ years) [Technical Report]. What Works Wellbeing. https://whatworkswellbeing.org/resources/loneliness‐conceptual‐review/

[bjc12358-bib-0044] McIntyre, J. C. , Worsley, J. , Corcoran, R. , Harrison Woods, P. , & Bentall, R. P. (2018). Academic and non‐academic predictors of student psychological distress: The role of social identity and loneliness. Journal of Mental Health, 27, 230–239. 10.1080/09638237.2018.1437608 29436883

[bjc12358-bib-0045] Muldoon, O. (2020). Collective trauma. In J. Jetten , S.D. Reicher , S.A. Haslam & T. Cruwys (Eds.), Together apart: The psychology of COVID‐19 (pp. 84–88). Thousand Oaks, CA: SAGE.

[bjc12358-bib-0046] Murad, M. H. , Katabi, A. , Benkhadra, R. , & Montori, V. M. (2018). External validity, generalisability, applicability and directness: A brief primer. BMJ Evidence‐Based Medicine, 23(1), 17–19. 10.1136/ebmed-2017-110800 29367319

[bjc12358-bib-0047] Niven, K. , Totterdell, P. , Stride, C. B. , & Holman, D. (2011). Emotion regulation of others and self (EROS): The development and validation of a new individual difference measure. Current Psychology, 30(1), 53–73. 10.1007/s12144-011-9099-9

[bjc12358-bib-0048] Palan, S. , & Schitter, C. (2018). Prolific.ac—A subject pool for online experiments. Journal of Behavioral and Experimental Finance, 17, 22–27. 10.1016/j.jbef.2017.12.004

[bjc12358-bib-0049] Peplau, L. A. , & Perlman, D. (1982). Perspectives on loneliness. In L. A. Peplau & D. Perlman (Eds.), Loneliness: A sourcebook of current theory, research and therapy (pp. 1–18). Hoboken, NJ: Wiley.

[bjc12358-bib-0050] Perlman, D. , & Peplau, L. A. (1998). Loneliness. In H. Friedman (Ed.), Encyclopedia of mental health (Vol. 2, pp. 571–581). Cambridge, MA: Academic Press.

[bjc12358-bib-0051] Ployhart, R. E. , & MacKenzie, Jr, W. I. (2015). Two waves of measurement do not a longitudinal study make. More Statistical and Methodological Myths and Urban Legends, 85–99.

[bjc12358-bib-0052] Preece, D. A. , Goldenberg, A. , Becerra, R. , Boyes, M. , Hasking, P. , & Gross, J. J. (2021). Loneliness and emotion regulation. Personality and Individual Differences, 180, 110974. 10.1016/j.paid.2021.110974

[bjc12358-bib-0053] Prolific Team (2021). Representative samples. Prolific. https://researcher‐help.prolific.co/hc/en‐gb/articles/360019236753‐Representative‐samples

[bjc12358-bib-0054] Roberts, R. E. , Lewinsohn, P. M. , & Seeley, J. R. (1993). A brief measure of loneliness suitable for use with adolescents. Psychological Reports, 72(3_suppl), 1379–1391. 10.2466/pr0.1993.72.3c.1379 8337350

[bjc12358-bib-0055] Russell, D. , Cutrona, C. E. , Rose, J. , & Yurko, K. (1984). Social and emotional loneliness: An examination of Weiss’s typology of loneliness. Journal of Personality and Social Psychology, 46, 1313–1321. 10.1037/0022-3514.46.6.1313 6737214

[bjc12358-bib-0056] Salo, A. , Junttila, N. , & Vauras, M. (2020). Social and emotional loneliness: Longitudinal stability, interdependence, and intergenerational transmission among boys and girls. Family Relations, 69(1), 151–165. 10.1111/fare.12398

[bjc12358-bib-0057] Sani, F. , Herrera, M. , Wakefield, J. R. H. , Boroch, O. , & Gulyas, C. (2012). Comparing social contact and group identification as predictors of mental health. British Journal of Social Psychology, 51, 781–790. 10.1111/j.2044-8309.2012.02101.x 22550954

[bjc12358-bib-0058] Sharman, L. S. , Dingle, G. , Baker, M. , Fischer, A. , Gračanin, A. , Kardum, I. , … Vanman, E. J. (2019). The relationship of gender roles and beliefs to crying in an international sample. Frontiers in Psychology, 10, 10.3389/fpsyg.2019.02288 PMC6795704 31649598

[bjc12358-bib-0059] Solomon, Z. , Bensimon, M. , Greene, T. , Horesh, D. , & Ein‐Dor, T. (2015). Loneliness trajectories: The role of posttraumatic symptoms and social support. Journal of Loss and Trauma, 20(1), 1–21. 10.1080/15325024.2013.815055

[bjc12358-bib-0060] Steffens, N. K. , Jetten, J. , Haslam, C. , Cruwys, T. , & Haslam, S. A. (2016). Multiple social identities enhance health post‐retirement because they are a basis for giving social support. Frontiers in Psychology, 7. 10.3389/fpsyg.2016.01519 PMC5065989 27799916

[bjc12358-bib-0061] Tajfel, H. , & Turner, J. C. (1979). An integrative theory of intergroup conflict. In W. G. Austin & S. Worchel (Eds.), The social psychology of intergroup relations (1st, pp. 33–37). Monterey, CA: Brooks/Cole.

[bjc12358-bib-0062] Tull, M. T. , Barrett, H. M. , McMillan, E. S. , & Roemer, L. (2007). A preliminary investigation of the relationship between emotion regulation difficulties and posttraumatic stress symptoms. Behavior Therapy, 38, 303–313. 10.1016/j.beth.2006.10.001 17697854

[bjc12358-bib-0063] Turner, J. C. , Hogg, M. A. , Oakes, P. J. , Reicher, S. D. , & Wetherell, M. S. (1987). Rediscovering the social group: A self‐categorization theory. Hoboken, NJ: Blackwell.

[bjc12358-bib-0064] Valtorta, N. K. , Kanaan, M. , Gilbody, S. , Ronzi, S. , & Hanratty, B. (2016). Loneliness and social isolation as risk factors for coronary heart disease and stroke: Systematic review and meta‐analysis of longitudinal observational studies. Heart, 102, 1009–1016. 10.1136/heartjnl-2015-308790 27091846 PMC4941172

[bjc12358-bib-0065] Van Den Brink, R. H. S. , Schutter, N. , Hanssen, D. J. C. , Elzinga, B. M. , Rabeling‐Keus, I. M. , Stek, M. L. , … Oude Voshaar, R. C. (2018). Prognostic significance of social network, social support and loneliness for course of major depressive disorder in adulthood and old age. Epidemiology and Psychiatric Sciences, 27, 266–277. 10.1017/s2045796017000014 28183368 PMC6998855

[bjc12358-bib-0066] Vanhalst, J. , Gibb, B. E. , & Prinstein, M. J. (2017). Lonely adolescents exhibit heightened sensitivity for facial cues of emotion. Cognition and Emotion, 31, 377–383. 10.1080/02699931.2015.1092420 26461799 PMC5985447

[bjc12358-bib-0067] Verzeletti, C. , Zammuner, V. L. , Galli, C. , & Agnoli, S. (2016). Emotion regulation strategies and psychosocial well‐being in adolescence. Cogent Psychology, 3(1), Article 1. 10.1080/23311908.2016.1199294

[bjc12358-bib-0068] Wakefield, J. R. H. , Bowe, M. , Kellezi, B. , Mcnamara, N. , & Stevenson, C. (2019). When groups help and when groups harm: Origins, developments, and future directions of the “Social Cure” perspective of group dynamics. Social and Personality Psychology Compass, 13(3), e12440. 10.1111/spc3.12440

[bjc12358-bib-0069] Walter, Z. (2017). Transitions through homelessness: The impact of psychosocial factors on wellbeing and outcomes in a homeless sample [PhD Thesis, The University of Queensland]. 10.14264/uql.2017.606

[bjc12358-bib-0070] Wang, J. , Lloyd‐Evans, B. , Giacco, D. , Forsyth, R. , Nebo, C. , Mann, F. , & Johnson, S. (2017). Social isolation in mental health: A conceptual and methodological review. Social Psychiatry and Psychiatric Epidemiology, 52, 1451–1461. 10.1007/s00127-017-1446-1 29080941 PMC5702385

[bjc12358-bib-0071] Wang, J. , Mann, F. , Lloyd‐Evans, B. , Ma, R. , & Johnson, S. (2018). Associations between loneliness and perceived social support and outcomes of mental health problems: A systematic review. BMC Psychiatry, 18(1), Article 1. 10.1186/s12888-018-1736-5 PMC5975705 29843662

[bjc12358-bib-0072] Weiss, R. S. (1974). The provisions of social relationships. In Z. Rubin (Ed.), Doing unto others (pp. 17–26). Hoboken, NJ: Prentice‐Hall.

[bjc12358-bib-0073] Wilkialis, L. , Rodrigues, N. , Majeed, A. , Lee, Y. , Lipsitz, O. , Gill, H. , … McIntyre, R. S. (2021). Loneliness‐based impaired reward system pathway: Theoretical and clinical analysis and application. Psychiatry Research, 298, 113800. 10.1016/j.psychres.2021.113800 33618235

[bjc12358-bib-0074] Wu, C.‐H. , & Yao, G. (2008). Psychometric analysis of the short‐form UCLA Loneliness Scale (ULS‐8) in Taiwanese undergraduate students. Personality and Individual Differences, 44, 1762–1771. 10.1016/j.paid.2008.02.003

[bjc12358-bib-0075] Zaki, J. , & Williams, W. C. (2013). Interpersonal emotion regulation. Emotion, 13, 803–810. 10.1037/a0033839 24098929

